# Kinannote, a computer program to identify and classify members of the eukaryotic protein kinase superfamily

**DOI:** 10.1093/bioinformatics/btt419

**Published:** 2013-07-31

**Authors:** Jonathan M. Goldberg, Allison D. Griggs, Janet L. Smith, Brian J. Haas, Jennifer R. Wortman, Qiandong Zeng

**Affiliations:** ^1^Broad Institute, 7 Cambridge Center, Cambridge, MA 02142, USA and ^2^Massachusetts Institute of Technology, 77 Massachusetts Avenue, Cambridge, MA 02139, USA

## Abstract

**Motivation:** Kinases of the eukaryotic protein kinase superfamily are key regulators of most aspects eukaryotic cellular behavior and have provided several drug targets including kinases dysregulated in cancers. The rapid increase in the number of genomic sequences has created an acute need to identify and classify members of this important class of enzymes efficiently and accurately.

**Results:** Kinannote produces a draft kinome and comparative analyses for a predicted proteome using a single line command, and it is currently the only tool that automatically classifies protein kinases using the controlled vocabulary of Hanks and Hunter [Hanks and Hunter (1995)]. A hidden Markov model in combination with a position-specific scoring matrix is used by Kinannote to identify kinases, which are subsequently classified using a BLAST comparison with a local version of KinBase, the curated protein kinase dataset from www.kinase.com. Kinannote was tested on the predicted proteomes from four divergent species. The average sensitivity and precision for kinome retrieval from the test species are 94.4 and 96.8%. The ability of Kinannote to classify identified kinases was also evaluated, and the average sensitivity and precision for full classification of conserved kinases are 71.5 and 82.5%, respectively. Kinannote has had a significant impact on eukaryotic genome annotation, providing protein kinase annotations for 36 genomes made public by the Broad Institute in the period spanning 2009 to the present.

**Availability:** Kinannote is freely available at http://sourceforge.net/projects/kinannote.

**Contact:** jmgold@broadinstitute.org

**Supplementary information:** Supplementary data are available at *Bioinformatics* online.

## 1 INTRODUCTION

Protein kinases are well-studied enzymes involved in the regulation of the majority of eukaryotic cellular processes. Mutations in protein kinases frequently cause human disease, and kinases have provided several drug targets (Johnson, 2009). Protein kinases act by transferring phosphate groups from ATP to the amino acid side chains of target proteins, a modification that often profoundly alters the biological activity of the target molecule. There are hundreds of types of protein kinases, which, despite their common mechanism, act specifically on diverse substrates and are themselves acted on by diverse regulators. The complete set of protein kinases, or kinome, encoded in an organism’s genome, has a profound impact on the biological properties of that organism. For example, the advent of the tyrosine kinase (TK) group, of kinases (protein kinase category abbreviations are in Supplementary Table S1) correlates with the rise of the metazoans (Manning *et al.*, 2008).

It is often possible to infer the function or specificity of a kinase discovered in a new organism by comparison with known examples, as many kinases are well conserved. The task of relating a new kinase to a known one has been facilitated by work on the classification and evolution of protein kinases (Hanks and Hunter, 1995; Manning *et al.*, 2002a) and KinBase (www.kinase.com). These studies show that most protein kinases belong to the eukaryotic protein kinase (ePK) superfamily, whose members share a common ancestry and fold. The ePK superfamily is divided into eight major groups, which are themselves divided into families and subfamilies (Supplementary Table S1), although not all families fall within one of the major groups. Most ePKs are recognized by similarity to hidden Markov profiles such as the Pkinase.hmm (http://pfam.sanger.ac.uk/), but members of several families are divergent and are often overlooked in searches targeted toward typical kinases. Although a core set of the ePKs is conserved across eukaryotes, this superfamily is plastic and prone to expansion (Anamika *et al.*, 2008; Artz *et al.*, 2011; Champion *et al.*, 2004; Eisen *et al.*, 2006; Manning *et al.*, 2008; Manning, 2011; Peixoto *et al.*, 2010; Srivastava *et al.*, 2010; Stajich *et al.*, 2010). The hierarchical structure of the classification system of Hanks, Hunter and Manning is well-suited to handle this plasticity, as it allows partial classification of newly discovered kinases and informatively describes relationships between similar but non-orthologous kinases.

A much smaller number of protein kinases, designated atypical protein kinases (aPKs), have either limited similarity to ePKs (protein kinase-like sequences, or PKLs), or differ from them altogether, and also differ from each other (Kannan and Neuwald, 2005; Leonard *et al.*, 1998; Scheeff and Bourne, 2005). Well-conserved aPKs have been annotated in several curated kinomes (Manning *et al.*, 2002b), and are included in KinBase (www.kinase.com).

The surge in the availability of genomic data has created a need to automate identification and classification of conserved and novel protein kinases. Current identification methods favor searches against protein kinase hidden Markov models (HMMs) from Pfam (Punta *et al.*, 2012) or Kinomer (Martin *et al.*, 2009), and searches against position-specific scoring matrices (PSSMs) from the Conserved Domain Database (Marchler-Bauer *et al.*, 2013). These methods effectively identify average kinases but are often unable to identify novel or divergent superfamily members; moreover, classification based on Pfam and Kinomer HMMs does not exceed the group level. Sequence similarity searches against a specialized database (e.g. KinBase) provide more complete classification of kinases from families represented in the database, but results for novel kinases may be difficult to interpret, and the diversity of the ePK superfamily does not lend itself to application of a universal score threshold. Orthology-based methods (Li *et al.*, 2003) can provide accurate classification, but their success hinges on the availability of appropriate reference data. An ortholog must be present and identified in the reference genome to make a classification in the target genome; thus, non-orthologous kinases will be overlooked. None of the aforementioned methods are able to generate an accurate kinome without manual curation by a knowledgeable user, and therefore are not viable solutions to high-throughput kinase annotation.

Here, we present Kinannote, an ePK identification and classification package that leverages a protein kinase HMM and similarity with known kinases to produce a high-quality draft kinome for a given gene set with a single command. Kinannote depends on two readily available third-party programs (BLAST and HMMER), and its input is a single fasta-formatted file, which may contain the complete set of predicted proteins from an organism, or as few as one sequence. The classifications produced by Kinannote are constructed according to the controlled vocabulary introduced by Hanks and Hunter (1995) and maintained at www.kinase.com. This allows information about related kinases to be directly mapped to newly classified kinases from the literature and resources such as ProKinO (Gosal *et al.*, 2011), the Protein Kinase Resource (Smith *et al.*, 1997), KinG (Krupa *et al.*, 2004) and PhosphoSite (Hornbeck *et al.*, 2012). Kinannote generates several files, including a table of kinases and their classifications and phylogenetic profiles of discovered kinases. We show that Kinannote performs well on four reference kinomes broadly representative of the eukaryotic tree-of-life (*Amphimedon queenslandica*, *Schizosaccharomyces pombe*, *Plasmodium falciparum* and *Giardia lamblia*) and describe its use to provide kinome annotations for 36 eukaryotic genomes released by The Broad Institute from 2009 through the time of publication (www.broadinstitute.org).

## 2 METHODS

### 2.1 Algorithm

**Fig. 1.**
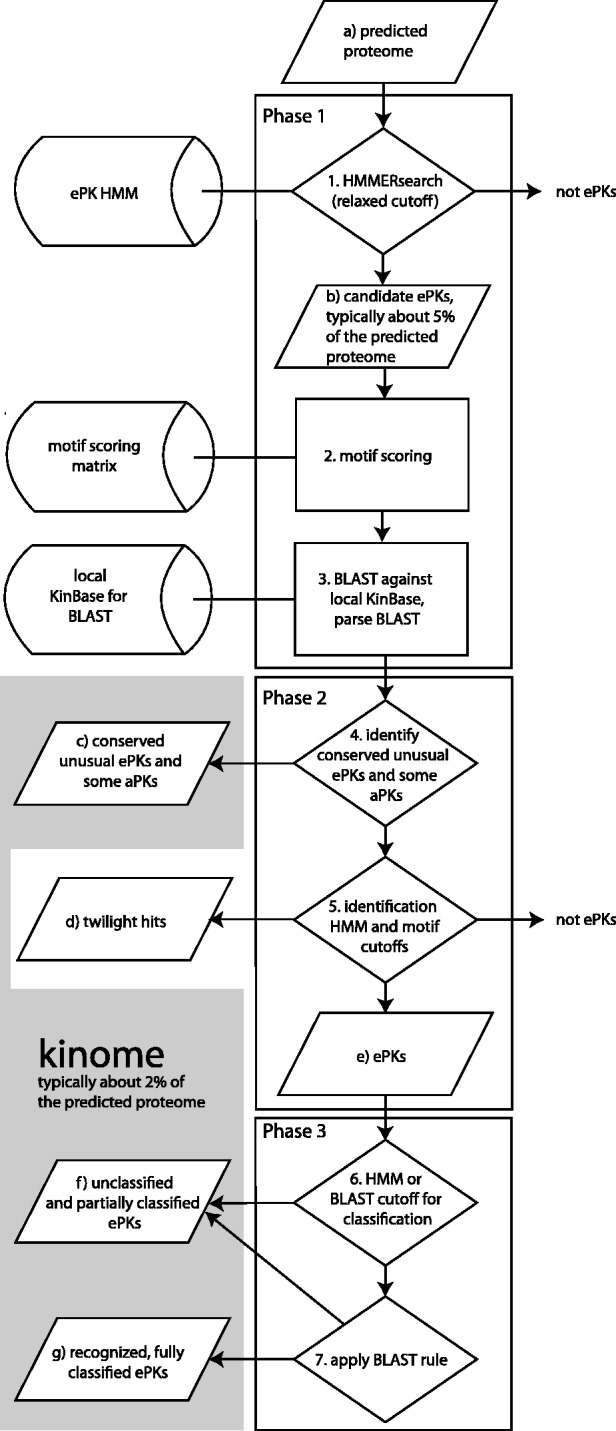
Algorithm used by Kinannote to produce a draft kinome from a predicted proteome. Computational steps are numbered and sequence sets are indicated by letters. The aggregate of sets comprising the draft kinome are indicated by the shaded area. Unclassified kinases may be species-specific or members of novel families; twilight hits are recorded but are not part of the draft kinome. ePKs, eukaryotic protein kinase superfamily members; aPKs, atypical protein kinases

Kinannote identifies and classifies protein kinases in three phases (Fig. 1); the first phase gathers information about the input sequences and defines a candidate set from which most non-kinases have been eliminated; this phase searches a predicted proteome, or subset thereof, with a protein kinase HMM and applies a relaxed cutoff that reduces the search space while retaining divergent kinases. The candidates are searched with a PSSM and against a local version of KinBase using BLAST, and the BLAST results are parsed. In phase 2, the BLAST results are used to identify conserved kinases with poor HMM scores. Once the unusual kinases have been identified, twilight hits and high-confidence protein kinases are identified using their PSSM scores and a more stringent HMM cutoff, resulting in a set of high-confidence ePKs. In phase 3, BLAST search results are applied to classify ePKs. These sequences are combined with the unusual protein kinases identified in phase 2, and the new kinome is compared with reference kinomes.

#### 2.1.1 Phase 1: First cut and gathering information

Kinannote uses an HMM derived from a manual alignment of the *Dictyostelium* kinome (Goldberg *et al.* 2006). The *Dictyostelium* branch-point on the eukaryotic tree is centrally located (Baldauf, 2003), resulting in a model sensitive to ePKs from diverse parts of the tree. This HMM is used in a HMMER search of the input protein set [HMMER 2.3.2 (Eddy, 1998)] using a relaxed cutoff (HMM E-value for candidate selection, Table 1; Fig. 1.1), resulting in a reduction of the search space for subsequent steps by ∼95% without loss of divergent kinases. The remaining sequences are referred to as candidates (Fig. 1b); their scores and alignments to the HMM are stored for later reference.

**Table 1.** Cutoffs used by Kinannote

Description	Affects	Optimized	Relaxed	Test Ranges
HMM E-value for candidate selection	Identification	100	200	
HMM score for kinase identification	Identification	−66	−200	−200, −100, −66, 1, 20, 100
Motif score for twilight hit identification	Identification	0.4	0.1	
HMM score for twilight hit identification	Identification	−173	−200	
BLAST E-value for identification of atypical protein kinases	Identification/Classification	10^−20^	10	
BLAST E-value for classification	Classification	10^−10^	1	1, 10^−10^, 10^−30^, 10^−80^, 10^−100^
HMM score for classification	Classification	−30	−200	
Number of consistent top BLAST hits for classification	Classification	3	1	1, 2, 3, 4, 5
Logarithm of BLAST E-value for subfamily classification	Classification	−30	1	

Protein kinase domains contain highly conserved substructures involved in catalysis, substrate binding and magnesium binding (Hanks and Hunter, 1995; Kannan and Neuwald, 2005). The identities of residues in catalytic substructures are often more constrained by functional requirements than by the need, distributed over a larger number of residues, to stabilize the protein (Fersht, 1985). As substitution matrices used by common profile-building methods are most influenced by the effects of residues on protein stability (Henikoff and Henikoff, 1992), profiles based on these methods may underestimate the relative importance of functional residues. To obtain a PSSM more sensitive to kinase subdomain motifs, we made a HMMER alignment of domains from curated kinases (www.kinase.com). Most residues observed in this alignment are ‘viable’, meaning that they occur in active kinases and are thus given equal weight in our PSSM.

Positions in the scoring matrix are weighted inversely to the sequence variability at the corresponding alignment position; therefore, for example, the position two residues upstream of the catalytic aspartate is highly weighted and has viable residues H and Y. Residues in candidate sequences receive scores based on their alignment positions if they contain viable residues, or scores of zero if they do not contain viable residues. Positional scores are summed to provide sequence scores, which are applied in phase 2 of the algorithm. For additional details on the PSSM, see Supplementary Document S1.

Candidates are searched with BLAST (Altschul *et al.*, 1997) against a local version of selected curated kinomes from KinBase downloaded on 23 December 2011 (Fig. 1.3). The set of curated kinomes used by Kinannote is indicated in Supplementary Table S2. The BLAST database contains both ePKs, for which the sequences of the kinase domains are used for reference, and aPKs, for which the entire protein sequences are used; the results of this search are applied later in the algorithm.

#### 2.1.2 Phase 2: Identification

*Identification of conserved unusual kinases and some aPKs:* Conserved ePKs with unusual sequences such as SLOB, SCYL1, Bub, Bud32 and Haspin tend to score poorly in searches using protein kinase HMMs. They are identified at this stage (Fig. 1.4) owing to the presence of similar kinases in KinBase. Atypical protein kinases were not used to build the HMM used for the HMMER search; however, several types of PKL aPKs, notably RIO and ABC1, are similar enough to ePKs (Kannan and Neuwald, 2005; Leonard *et al.*, 1998; Scheeff and Bourne, 2005) to enter the candidate set and are identified by similarity to relatives in KinBase. Unusual protein kinases and aPKs thus identified are included in the kinome (Fig. 1c).

*Identification of typical kinases and twilight hits:* Once conserved unusual kinases and PKL aPKs have been identified, non-kinases may be removed from the candidate set without loss of poorly scoring conserved kinases (Fig. 1.5). Sequences scoring below the PSSM cutoff (Motif score for twilight hit identification, Table 1) are dropped, and sequences above this cutoff and below the ‘HMM score for kinase identification’ (Table 1) are identified as twilight hits (Fig. 1d). The remaining sequences are considered ePKs, added to the kinome (Fig. 1e) and classified. Twilight hits, designated ‘protein kinase subdomain-containing proteins’, are not part of the final kinome but may provide seed sequences for delineating novel PKL families.

#### 2.1.3 Phase 3: Classification

*Unclassified kinases: *To avoid misclassifying potentially novel, divergent kinases, or kinases with incorrect gene models, Kinannote refrains from classifying poorly scoring ePKs. Sequences with E-values greater than the ‘BLAST E-value for classification’ and scores below the ‘HMM score for classification’ (Table 1) are not classified. The suggested gene product name for these sequences is ‘serine/threonine protein kinase’, and they are flagged in files produced by Kinannote to facilitate their further curation. Sequences for which the group level classifications of the top BLAST hits are inconsistent (see later in the text) are also not classified.

*Full classification:* Kinannote classifies the remaining ePKs into groups, families and subfamilies (Fig. 1.7) using top BLAST hits. The algorithm requires that a set number of consecutive top BLAST hits agree to provide a classification (Number of consistent BLAST hits, Table 1); as there are three levels of classification, hits may agree, or not agree, at the group, family, or subfamily levels. Full classifications are made if the top BLAST hits agree at the same classification depth as the reference kinase, which may be classified either to the group/family level or the group/family/subfamily level. Kinannote allows the number of consecutive top BLAST hits that are considered in making classification decisions to be set by the user.

*Partial classification and uncertainty:* Protein kinases discovered in new genomes often do not fit into existing families or subfamilies but are evolutionarily related to known groups or families. Kinannote partially classifies these kinases if possible. If the consecutive top BLAST hits for a sequence are consistent at the group level, but not at the family level, the sequence is partially classified to that group. If the top BLAST hits against a sequence agree at the group and family level, but not at the subfamily level, the sequence is classified to that group and family. Partial classification may also result for kinases with incomplete gene models or in species that are evolutionarily divergent from those in the reference database.

*TK classification:* The TK group is thought to have arisen on the branch that gave rise to metazoans after divergence from the fungal branch (Manning *et al.*, 2008); classification of earlier-evolving TK group members thus requires manual verification. Kinannote takes a conservative approach toward TK classification in species that are not metazoans or their close relatives. In non-metazoans, kinases for which the best BLAST hits against the reference database are TK group members are classified as tyrosine kinase-like (TKL) and flagged in the draft kinome table produced by Kinannote. Kinannote treats input as non-metazoan by default; the user must run Kinannote with the ‘-m’ flag in the command line to classify TK group members normally in metazoans and their relatives (Supplementary File S1 and online documentation at sourceforge.net/projects/kinannote).

The union of fully and partially classified ePKs, unclassified ePKs, divergent ePKs and aPKs (Fig. 1 sets g, f, c, respectively) is referred to as the draft kinome. Kinannote records the draft kinome with its associated search scores, labels twilight hits as ‘protein kinase subdomain-containing proteins’ and flags rejected candidates as ‘subthreshold’. This file is used for post-run processing.

### 2.2 Summary, comparative kinomics, the draft kinome and gene product names

Kinannote provides extensive metadata and contextual information about the new draft kinome (Supplementary Table S3). A compact summary table provides a total kinase count, and counts for the group and unclassified categories. If the input is a whole proteome, then the percentage of kinases in the predicted protein set is given, and a list of missing core kinases is provided, where the core is determined by presence in the set of references kinomes from *Dictyostelium discoideum*, *Saccharomyces cerervisiae*, *Drosophila melanogaster*, *Caenorhabdidtis elegans* and *Homo sapiens*. The analyses in this file allow the completeness of the kinome to be assessed, which serves as a proxy for the coverage of the input proteome. A phylogenetic comparison of the new kinome (or kinome subset) and reference kinomes is provided in a separate table. The missing core kinases identified in these tables provide a basis for hole-filling and comparative analysis. A list of classification-based gene product names and a summary of the parameters used in the run are also provided. Kinannote produces a file containing the parameters that were used for the latest run. Parameters in this file may modified by the user, and the file may be renamed, and read using the command line ‘-p’ flag (Supplementary File S1 and online documentation at sourceforge.net/projects/kinannote).

Kinannote provides output with details about each identified kinase (the draft kinome table), twilight hits and rejected candidates. This table includes a column indicating the classification depth of each kinase: ‘0’ if the kinase is unclassified, ‘1’ if it is classified only to the group level, and ‘2’ or ‘3’ if it is classified to the family or subfamily levels, respectively. The classification fields associated with unclassified kinases remain empty in this table, but they are identified as ‘unclassified’ in another column. Kinases with classification depths of 0 or 1 may be members of novel families. The classification depth number allows potentially novel kinases and conserved kinases to be separated into two categories, providing a good foundation for further curation of potentially novel kinases. Members of PKL expansions contain recognizable kinase motifs but score poorly against profiles built from typical kinases (Leonard *et al.*, 1998). These sequences are identified using Kinannote’s PSSM (see Section 2); they are not included in the draft kinome, but they are labeled ‘protein kinase subdomain-containing proteins’ in the draft kinome table. In genomes where novel PKL families occur, this list of twilight hits may provide a starting point for further curation.

Kinannote provides a list of gene product names based on classification results. Classified kinases are named ‘group/family/subfamily protein kinase’ using the controlled vocabulary maintained at KinBase: the family and subfamily fields may be empty, depending on the classification result. Unclassified kinases are named ‘serine/threonine protein kinase’.

### 2.3 Test cases

Kinannote was tested on the full sets of predicted proteins from four species for which curated kinomes are available: *A.queenslandica* (Srivastava *et al.*, 2010), *G.lamblia* (Manning *et al.*, 2011), *P.falciparum* (Talevich *et al.*, 2011) and *S.pombe* (Rhind *et al.*, 2011) (Supplementary Table S2). These species were chosen because they represent different parts of the eukaryotic tree, their kinomes are comprehensive and well-classified using the controlled vocabulary of Hanks and Hunter (1995) and www.kinase.com, and they are not part of the reference database used by Kinannote for identification or classification. The curation levels of these test kinomes are adequate evaluating kinase prediction and classification algorithms, but they are not curated to the level of many of the kinomes used in the reference database from KinBase, particularly those of *H.sapiens*, *D.melanogaster*, *C.elegans*, *S.cerevisaie* and *D.discoideum*, for which most kinases received individual attention. In addition, the fragmentation of the *A.queenslandica* assembly results in many partial gene models that are difficult to curate definitively.

For comparison, tests were performed using protein kinase HMMs downloaded from the Kinomer resource at www.compbio.dundee.ac.uk/kinomer (Martin *et al.*, 2009). Reference proteomes were searched with Kinomer HMMs using the recommended cutoff score of 20, and kinases were classified according to the group of the best scoring HMM hit.

### 2.4 Receiver operating characteristic analysis

To evaluate the performance of Kinannote with respect to the curated datasets using receiver operating characteristic (ROC) analysis (Metz, 2006), it must be determined whether a prediction is a true positive (TP), true negative, false positive (FP) or false negative (FN). For protein kinase identification, this is straightforward (a sequence either is or is not an ePK). Similarly, it is easy to determine whether a classification provided by Kinannote exactly matches that of the curated kinase.

Assessing the results for partially classified kinases is more complicated. If the reference sequence is classified to the subfamily level, and Kinannote correctly classifies it to the group and family levels and leaves the subfamily null, we define this as TP for partial classification and FN for full classification. If Kinannote correctly classifies the group and leaves both family and subfamily null, this is also counted as TP for partial and FN for full classification, respectively. If Kinannote incorrectly assigns the subfamily, we define this as FP even if the group and family match the reference because misclassification negates correct partial classification in terms of the value of the annotation to a user. If Kinannote provides no classification for a classified reference kinase, the partial classification result is FN. Finally, if Kinannote and the reference agree that the sequence is an unclassified kinase, or is not a kinase, the partial classification is true negative. Similar logic is applied when the reference kinase is only classified to the family level. The results of application of the Kinomer HMMs to test proteomes were similarly categorized.

For kinase identification and classification the false-discovery rate is FDR = FP/(TP + FP), and the true-positive rate (TPR), sensitivity or recall is TPR = TP /(TP + FN), where TP + FN is P, the number of reference positives. The precision is PPV = TP/(TP + FP), and the F-score is 2*PPV*TPR/(PPV + TPR). A single sequence may have different characteristics, e.g. a TP for identification may be an FN for classification. For all ROC analyses, the total number of kinases in the reference set is used as the value of P.

### 2.5 Draft kinome production

Supplementary Table S2 includes 36 species for which draft kinome annotations were produced by Kinannote, or for which curated kinome annotation was assisted by Kinannote. These kinomes have been incorporated into annotations made public by the Broad Institute in the period from 2009 to the present (www.broadinstitute.org). Most of these annotations are also available at NCBI (http://www.ncbi.nlm.nih.gov/). A cladogram used to illustrate phylogenetic relationships among species used in this study are based on published phylogenetic analyses (Baldauf, 2003; Desjardins *et al.*, 2013; Duplessis *et al.*, 2011; McLaughlin *et al.*, 2009; Paps *et al.*, 2013; Rhind *et al.*, 2011; Srivastava *et al.*, 2010; White *et al.*, 2008).

## 3 RESULTS AND DISCUSSION

### 3.1 Optimizing parameters for kinase identification

**Fig. 2.**
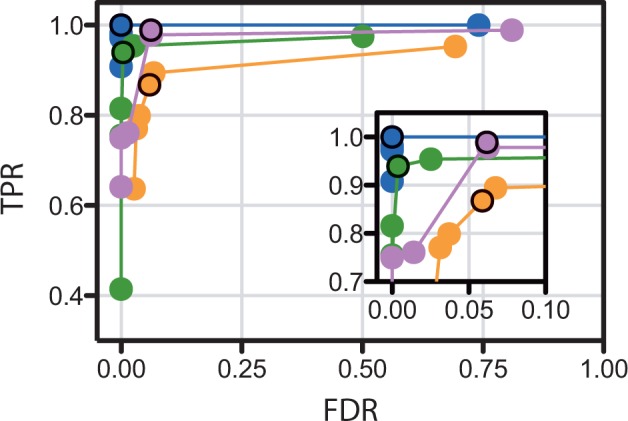
Receiver-operator characteristic (ROC) curves for identification of protein kinases in reference kinomes by Kinannote. The cutoff score (HMM score for kinase identification, Table 1) of results from a search of the comprehensive predicted gene set against a protein kinase hidden Markov model (Fig. 1.1, cutoff applied at Fig. 1.5) was varied from stringent (left side of range) to permissive (right side of range). Blue, *S.pombe*; orange, *A.queenslandica*; green, C. *G.lamblia*; purple, *P.falciparum*. The upper left corner of the plot is expanded in the inset. Points representing the optimum setting of −66 are circled. TPR, true positive rate; FDR, false-discovery rate

The parameter with the greatest impact on kinase identification is the cutoff score from the search of the input sequences against the *Dictyostelium* kinome-based HMM (HMM score for kinase identification, Table 1). This cutoff, applied at Figure 1.5, is more stringent than initial relaxed cutoff applied at Figure 1.1. Test runs were produced for ROC analysis (Fig. 2) by varying the cutoff value from stringent to permissive. The ROC curves resulting from variation of this parameter allow the optimal cutoff score to be identified (Table 1). Similar analyses (not shown) allowed selection of default values for the other parameters affecting kinase identification (Table 1).

### 3.2 Optimizing parameters for kinase classification

**Fig. 3.**
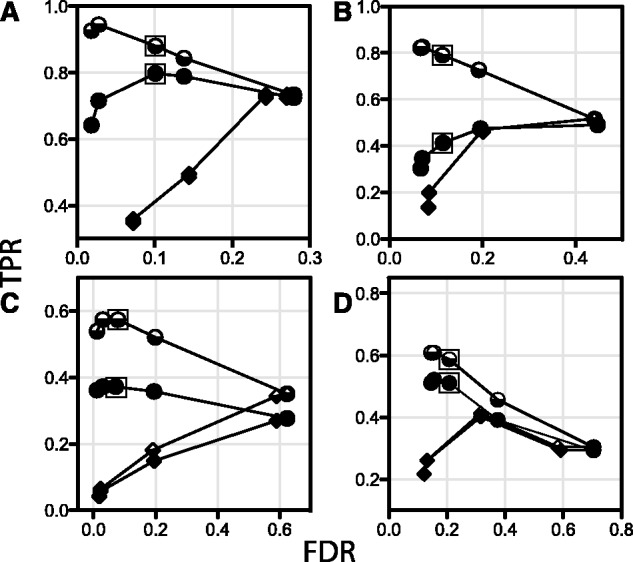
ROC analysis to identify optimum settings for classification by Kinannote and compare classification methods. Circles: effect on classification of varying the number of consistent consecutive top BLAST hits of candidate kinases against the reference database. The BLAST search is described in Figure 1.3, and the criterion is applied at Figure 1.7. The number of consistent hits required for classification ranged from 5 (left side of range) to 1 (right side of range). Partial classification is indicated by half-filled symbols, and full classification is indicated by filled symbols. The points obtained at three consistent consecutive hits are indicated with boxes. This setting was chosen as the default value for Kinannote because it provides a good compromise between sensitivity and precision. Diamonds: effect on classification of varying the E-value cutoff from the BLAST search (Fig. 1.6) from 1 × 10^−100^ (left side of range) to 1 (right side of range). As aforementioned, partial and full classification are indicated by half-filled and filled symbols, respectively. The test genomes are A, *S.pombe*; B, *A.queenslandica*; C, *G.lamblia*; and D, *P.falciparum*. TPR, true-positive rate; FDR, false-discovery rate

The parameters with the greatest impact on classification are from the BLAST search of kinase candidates against KinBase (Fig. 1.3). These parameters, applied at Figure 1.6 and 1.7, respectively, are the E-value of the top hit, and the number of consistent top hits required for classification (BLAST E-value for classification, number of consistent top BLAST hits for classification, respectively, Table 1). Test Kinannote runs for ROC analysis (Fig. 3) were produced with these parameters varied from stringent to permissive. In general, the TPR is expected to rise as the FDR falls, but for the ROC curves where the number of consistent top BLAST hits is varied, the TPR rises, then falls, as FDR rises. This occurs because kinases that are correctly placed into families when a greater number of consistent top BLAST hits were required are misclassified to incorrect subfamilies when fewer consistent top hits are used. The fall in TPR as the BLAST cutoff E-value rises in *P.falciparum* (Fig. 3D) results from the presence of unclassified kinases in the reference kinome, which are misclassified at permissive cutoffs.

A striking result apparent in Figure 3 is the better classification obtained using three or four consistent top BLAST hits as a limiting condition rather than any BLAST E-value cutoff. This is because assignment by top BLAST hits allows classification of loosely affiliated families, such as the SRPK family. A BLAST E-value cutoff relaxed enough to allow classification of SRPKs would result in misclassifications elsewhere in the kinome. The reference database must have sufficient depth for classification by number of consistent top BLAST hits to succeed, suggesting that the performance of Kinannote with respect to classification may be improved by adding breadth and depth to the reference database.

An important conclusion from the ROC curves for kinase identification (Fig. 2) and classification (Fig. 3) is that the optimal settings are fairly consistent from species to species. This means that the set of optimal parameters identified here (Table 1) and built into Kinannote should give similar results when the program is applied to divergent species.

### 3.3 Performance evaluation

With the optimal parameters selected, Kinannote’s performance on the four curated test genomes may be evaluated. Kinannote is the only tool currently available that produces a draft kinome using a single line command. To obtain a measure of comparison with existing resources, we downloaded the suite of protein kinase HMMs from Kinomer and used them to identify protein kinases and classify them to the group level (see Section 2); comparison of Kinannote and Kinomer is thus restricted to identification and group-level classification.

#### 3.3.1 Kinase identification performance

**Fig. 4.**
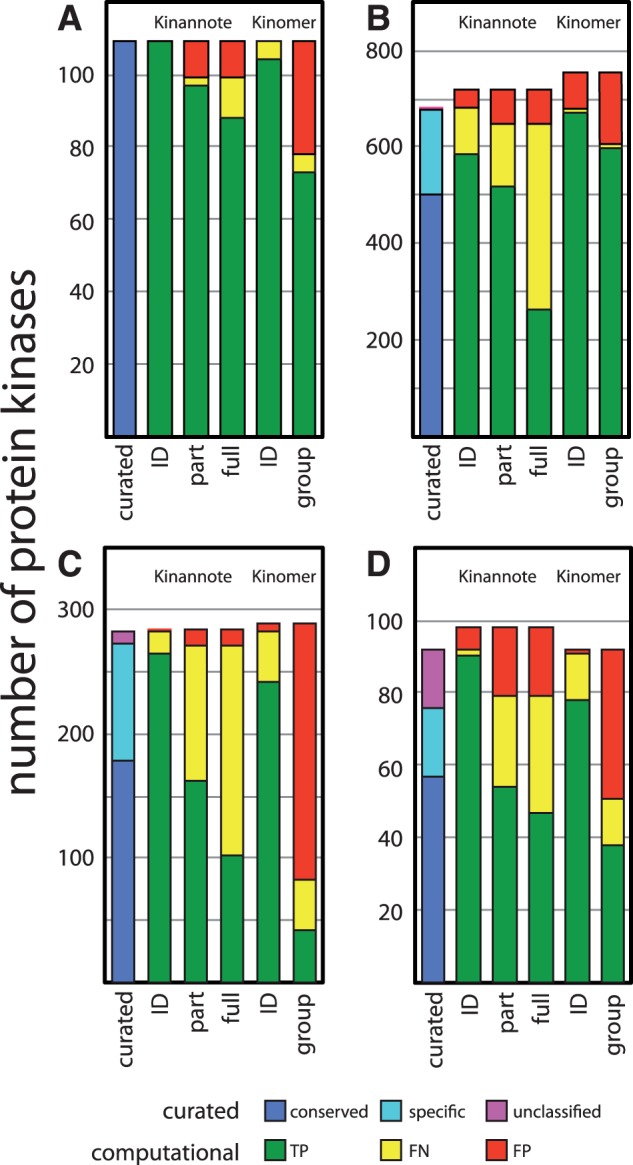
Performance of Kinannote and Kinomer HMMs on curated kinomes. Kinannote identification, partial classification and full classification results are shown in the second, third, and fourth columns, respectively. Kinomer identification and group-level classification are shown in the fifth and sixth columns, respectively. Curated kinase counts are shown in the first columns. For Kinannote and Kinomer, the columns are divided into TP, FN, FP categories; for the curated kinomes, the columns are divided into conserved, species-specific and unclassified categories. The colors associated with these categories are indicated in the key below the figure. The curated kinomes are from A, *S.pombe*; B, *A.queenslandica*; C, *G.lamblia*; and D, *P.falciparum*

Kinannote sensitively and precisely identifies protein kinases (Fig. 4, compare kinases identified by Kinannote in the second columns with the curated kinomes in first columns). Kinannote perfectly retrieves the *S.pombe* kinome. Kinannote identifies more kinases than the Kinomer HMMs in *S.pombe*, *P.falciparum* and *G.lamblia* (Fig. 4, compare green regions of the second and fifth columns); these additional kinases include conserved kinases with unusual sequences and species-specific kinases. A case-by-case examination of false-positive identification calls made by Kinannote indicates that a majority result from FN calls in test kinomes. Further examination of FP calls is provided in Supplementary Document S1. Kinomer finds more kinases in *A.queenslandica* than does Kinannote. These additional kinases, many of which are small fragments, belong to a large TK-expansion in the sponge; the TK-based HMMs of Kinomer are more sensitive to divergent members of this group than the HMM used by Kinannote. Adaptation of these HMMs may improve future versions of Kinannote.

The sensitivity, precision and the F-score for kinase identification and classification of the four curated kinomes are given in Table 2. These values were averaged to provide a summary of the performance of Kinannote and our application of the Kinomer HMMs. Overall, Kinannote identifies reference kinases with an average sensitivities and precisions of 94.4 and 96.8%, respectively. The corresponding values for the Kinomer HMMs are 91.6 and 96.7%. The average sensitivity of Kinannote is 2.8% greater than that of Kinomer. This results from the ability of Kinannote to identify BUB, Bud32, Haspin, SCYL and SLOB kinases, which are widely conserved, but whose sequences diverge from those of most kinases. Additional sensitivity results from Kinannote’s greater ability to identify potentially novel divergent kinases.

**Table 2.** Performance of Kinannote and comparison with results from application of Kinomer HMMs

Spe.	Lev.	Kinannote			Kinomer HMMs		
		S	P	F	S	P	F
Sp	ID	100.0	100.0	100.0	96.3	100.0	98.2
Sp	PT	98.0	90.7	89.8	93.6	70.2	68.1
Sp	FL	88.9	89.8	84.7			
Aq	ID	85.8	93.9	86.9	98.7	90.7	90.2
Aq	PT	79.9	88.1	78.6	98.6	80.3	79.9
Aq	FL	41.0	78.6	46.2			
Gl	ID	94.0	99.6	96.7	85.8	97.2	90.2
Gl	PT	60.1	93.1	70.9	51.2	16.9	11.8
Gl	FL	37.9	90.4	50.8			
Pf	ID	97.8	93.8	92.8	85.7	98.7	91.3
Pf	PT	68.4	74.0	60.4	74.5	48.1	41.3
Pf	FL	59.5	71.2	53.4			
							
Ave.	ID	94.4	96.8	94.1	91.6	96.7	92.5
Ave.	PT	76.6	86.5	74.9	79.5	53.9	50.3
Ave.	FL	56.8	82.5	58.8			

*Note*: Spe., species; Lev., classification level; S, sensitivity; P, precision; F, F-score; Sp, *Schizosaccharomyces pombe*; Aq, *Amphimedon queenslandica*; Gl, *Giardia lamblia*; Pf, *Plasmodium falciparum*; ID, kinase identification; PT, partial classification; FL, full classification*.*

#### 3.3.2 Kinase classification performance

Protein kinases may be fully or partially classified into groups, families and subfamilies in a manner similar to enzyme classification by EC number. A full classification exactly matches a category in KinBase, whereas a partial classification matches at the group level, or at the group and family levels in cases where that family is further divided into subfamilies. Kinannote effectively fully and partially classifies more kinases than does our application of the Kinomer HMMs (Fig. 4A–D, green regions of the third and fourth columns). Kinannote takes a conservative approach to reflect uncertainty in classification, withholding full, and sometimes partial classification when the top BLAST hits against the reference database are inconsistent, or the BLAST E-value of the best hit is above threshold (Table 1). Such classifications are considered FNs for full or partial classification (Fig. 4A–D, yellow regions of the third and fourth columns); they are included in the kinome, and they are TPs for kinase identification.

A case-by-case examination reveals two causes for the majority of false-positive classification calls made by Kinannote: FP calls against *S.pombe* are minor, resulting from nomenclature updates and subfamily additions to the fungal reference kinomes after curation of the fission yeast. The other test species are distantly related to the reference species and have species-specific kinase expansions. In some instances, Kinannote classified species-specific kinases based on the most closely related reference kinases. Errors of this kind may be ameliorated by extension of the reference database. Further examination of FP calls is provided in Supplementary Document S1.

Overall, Kinannote partially classifies the reference kinomes with average sensitivities and precisions of 76.6 and 84.5%, respectively (Table 2). The corresponding values for classification of kinases to the group level by application of the Kinomer HMMs are 79.5 and 53.9%, respectively. The relatively low precision of partial classification by Kinomer (Fig. 4, fifth columns) is the result of placement of unaffiliated kinases (kinases from the ‘Other’ group) into one of the major groups. The average sensitivity and precision values for full classification by Kinannote are 56.8 and 82.5%, respectively. If species-specific kinases (cyan blocks in Fig. 4, first columns) are excluded from the calculation, the average sensitivity for full classification by Kinannote rises to 71.5%.

Taken together, these results show that Kinannote identifies and fully or partially classifies a wide range of protein kinases with good sensitivity and precision. This, in addition to Kinannote’s ease of use, fulfills the need for a reliable high-throughput method for kinome annotation.

### 3.4 Impact of kinannote on eukaryotic genome annotation

As of early 2013, Kinannote, or a combination of Kinannote and manual curation, have been used to annotate the kinomes of 36 genomes produced by the Broad Institute (Supplementary Table S2). An overview of 25 of these new kinomes, representing a wide spectrum of eukaryotes, is given in Supplementary Document S1 and Supplementary Figure S1. The accession numbers and phylogenetic profiles for these kinomes are provided in Supplementary Tables S4 and S5, respectively.

### 3.5 Future directions

Addition of the test kinomes used for evaluation in this report to the reference kinomes used by Kinannote will allow better classification of Fungal, Diplomonada, Apicomplexan and basal metazoan kinases. Inclusion of new kinomes from Dermatophytes (Martinez *et al.*, 2012) and Apicomplexans (Miranda-Saavedra *et al.*, 2012; Talevich *et al.*, 2011) and nematodes (Desjardins *et al.*, 2013) will provided better depth and allow classification of members of newly described kinase families present in these clades. Results from recent comparative genomic studies (Beakes *et al.*, 2012; Cuomo *et al.*, 2012; Neafsey *et al.*, 2012) may be targeted for kinome curation to provide better coverage of undersampled eukaryotic groups.

Many aPK families are described by specific Pfam HMMs; inclusion of additional searches with these HMMs will allow most aPKs to be annotated. The addition of searches against Kinomer HMMs will improve sensitivity toward TK-group expansions in metazoans and pre-metazoans. Updated versions of Kinannote will be available at http://sourceforge.net/projects/kinannote.

We will continue to use Kinannote to provide high-quality automated kinome annotations for genomes sequenced at the Broad Institute. We will also use Kinannote to generate data for in-depth genomic and comparative biological studies, for the study of protein kinase and signal transduction pathway evolution and to identify potential drug and diagnostic targets in pathogenic species.

## Supplementary Material

Supplementary Data
