# Supplementary material for: Kinannote, a computer program to identify and classify members of the eukaryotic protein kinase superfamily
Source: Bioinformatics. 2013 Jul 31;29(19):2387–94. doi: 10.1093/bioinformatics/btt419 (PMC3777111; doi:10.1093/bioinformatics/btt419)
Supplement: Supplementary Data [file supp_29_19_2387__index.html]

Kinannote, a computer program to identify and classify members of the eukaryotic protein kinase superfamily — Kinannote, a computer program to identify and classify members of the eukaryotic protein kinase superfamily — Kinannote, a computer program to identify and classify members of the eukaryotic protein kinase superfamily — Supplementary Data 

# Kinannote, a computer program to identify and classify members of the eukaryotic protein kinase superfamily

## Supplementary Data

files

**Files in this Data Supplement:**

- Supplementary Data - zip file
