# Supplementary material for: Kinannote, a computer program to identify and classify members of the eukaryotic protein kinase superfamily
Source: Bioinformatics. 2013 Jul 31;29(19):2387–94. doi: 10.1093/bioinformatics/btt419 (PMC3777111; doi:10.1093/bioinformatics/btt419)
Supplement: Supplementary Data [file supp_btt419_suppl_data.zip › Supplemental_Document_S1.docx]

**Supplemental Document S1, Supplemental Figure S1, Supplemental File S1, and Supplemental Tables S1, S2, S3, S4 and S5 for:**

**Kinannote, a computer program to identify and classify members of the eukaryotic protein kinase superfamily**

Jonathan M. Goldberg^1,*^, Allison Griggs^1^, Janet L. Smith^2^, Brian Haas^1^, Jennifer Wortman^1^ and Qiandong Zeng^1^

^1^Broad Institute, 7 Cambridge Center, Cambridge, MA 02142

^2^Massachusetts Institute of Technology,

^*^To whom correspondence should be addressed.

Supplemental Document S1 2

Development of a Position-Specific Scoring Matrix for the Protein Kinase Domain 2

Investigation of False-Positive Kinannote Calls 3

Overview of Impact of Kinannote on Eukaryotic Genome Annotation 3

Supplemental Figure S1 5

Supplemental File S1. Compressed Kinannote Source Code, Libraries and Documentation Separate File

Supplemental Tables Separate File

S1. Abbreviations Separate File

S2. Reference and Test Kinomes Separate File

S3. Kinannote Output Separate File

S4. Selected Kinomes Annotated at The Broad Institute Separate File

S5. Phylogenetic Profile of Selected Kinomes Annotated at The Broad Institute Separate File

References 6

# Supplemental Document S1

## Development of a Position-Specific Scoring Matrix for the Protein Kinase Domain

In order to recognize conserved motifs within kinase candidate sequences, a position-specific scoring matrix (PSSM) was generated using a *Dictyostelium* kinome hidden Markov model (HMM) (Goldberg, et al. 2006) and sequences from KinBase, the protein kinase database from www.kinase.com. This matrix is built from the entire kinase domain in order to remove bias in the selection of regions.

Kinase domains from KinBase were aligned to the HMM, and the set of viable residues was computed for each position in the alignment using a cumulative binomial distribution function (binomial CDF). The expected frequency for each residue is

$$p_{residue}=\frac{instances of residue}{all instances of all residues}$$

where the number of observed instances of a residue at a position, *x*, is calculated from the alignment. A residue is considered viable if

$$1.0=F\left( x;n,p_{residue} \right)=Pr\left( X\leq x \right)= \sum_{i=0}^{\left\lfloor x \right\rfloor} \binom{n}{i}{p_{residue}}^{i}\left( 1-p_{residue} \right)^{n-i}$$

where *n* is the number of sequences. A threshold of 1.0 is selected so as to include all well-conserved kinase motifs in the set of viable residues. This set, for each position in the alignment, is used to create a regular expression composed of viable residues for the matrix. Since viable residues occur in curated kinases they are weighted equally in the regular expression; thus, rare viable residues are more highly weighted in the matrix than they are in the HMM.

Each position in the matrix is assigned a weight corresponding to its conservation in the alignment using normalized ratios of observed-to-expected-instances of both residues and alignment gaps. The score for a given residue at a given position can be expressed as

$${score}_{pos}=\frac{\frac{\sum_{i=1}^{vres} \frac{{observed}_{{pos}_{i}}}{{expected}_{{pos}_{i}}}}{\frac{{gap}_{pos}}{{gap}_{expected}}}}{\sum_{j=1}^{pos} \frac{\sum_{k=1}^{res} \frac{{observed}_{j_{k}}}{{expected}_{j_{k}}}}{\frac{{gap}_{j}}{{gap}_{expected}}}}, vres=\{viable residues\}$$

The score for a given sequence is

$$\sum_{i=1}^{pos} {score}_{i}$$

## Investigation of False-Positive Kinannote Calls

*Identification*. The Kinannote run on the predicted proteins of the *Amphimedon queenslandica* genome resulted in 37 false-positive kinase identification calls (Figure 4A). A case-by-case review revealed that twenty six resulted from false-negative calls in the curated kinome, and eight resulted from borderline cases in which sequences contained partial matches to protein kinase HMMs; just three cases were clear false-positive calls. The six false-positives calls in *Plasmodium falciparum* (Figure 4D) contained poly-asparagine regions, suggesting that application of a low-complexity filter to Kinannote or the reference kinomes might improve selectivity. There was one in false positive identification call in *Giardia lamblia* resulting from a sequence with a bitscore of 31.5 from a search against the Pkinase HMM; examination of the alignment of this sequence with the HMM consensus indicated was not a protein kinase. There were no false positive identification calls in *Schizosaccharomyces pombe.*

*Classification*. The false positive classification calls the Kinannote run on proteins encoded in the *Schizosaccharomyces pombe* genome result from minor differences in nomenclature introduced by an update in the reference database since curation of the *Schizosaccharomyces pombe* kinome. Most false-positive classification calls in *Amphimedon queenslandica* result from kinases which are missing from (see above) or misclassified in the curated set. The remaining incorrect calls result from placement of novel *Amphimedon*-specific TKL group kinases into related *Dictyostelium* families. Most false positive calls on the protein set from the *Giardia lamblia* genome result from placement of novel *Giardia*-specific NEK kinases into the conserved NEK2 family. Six false positive calls in *Plasmodium falciparum* result from incorrect identification calls described above, the majority of others reflect instances in which the classification by Kinannote appears to be better, or on par with the curated call.

## Overview of Impact of Kinannote on Eukaryotic Genome Annotation

Kinannote is currently used to provide standardized protein kinase gene product names for eukaryotic genomes annotated at the Broad Institute. Kinase names are formatted as ‘group/family/subfamily protein kinase’, where the classification is provided at the maximum granularity that can be assigned with confidence; unclassified protein kinases are named ‘serine/threonine protein kinase’. This controlled vocabulary allows users to access relevant literature, facilitates comparative analyses of kinomes, and identifies kinases that are novel and require additional curation.

As of early 2013 Kinannote, or a combination of Kinannote and manual curation, have been used to annotate the kinomes of 37 genomes produced by the Broad Institute (Supplemental Table S2). The broad outlines of 25 of these new kinomes, representing a wide spectrum of eukaryotes, are shown in Supplemental Figure S1 along with kinomes from KinBase and species used for evaluation. The accession numbers and phylogenetic profiles for these kinomes are provided in Supplemental Tables S3 and S4, respectively. These data provide significant new information on kinome evolution across the eukaryotes. A comprehensive analysis thereof is beyond our current scope, but it is appropriate to discuss briefly kinome sizes, and the size and distributions of major groups in order to highlight the potential impact of high-throughput kinome annotation. The CK1 group (regulation of general functions and vesicle sorting), and RGC and TK groups (detection and processing of external signals) are greatly expanded in *Caenorhabditis elegans* (Manning, et al., 2011). These groups are much smaller in the parasitic filarial worms *Loa loa* and *Wuchereria bancrofti*, suggesting that their increased size in *Caenorhabditis* *elegans* may be a response to the complex soil environment inhabited by the free-living nematode. Farther down the cladogram, the kinome of the rosette-forming choanoflagellate *Salpingoeca rosetta* is observed to be more than twice as large than that of the solitary *Sphaeroforma arctica*, suggesting a dramatic increase in signaling complexity between the branch points of the solitary Ichthyosporea and the social Choanoflagellata. Examination the metazoan and pre-metazoan portion of the figure supports the hypothesis that the receptor-guanylate cyclase (RGC) group is specific to metazoans. Members of this group contain catalytically inactive kinase domains involved in sensory processes in specialized cells, which relay signals to adjacent cells in multicellular organisms.

Examination of the fungal portion of the figure reveals a consistent core kinome of about 100 members. In addition to this core, the Onygenales, most notably the Pezizomycota, and *Coprinopsis cinerea*, a complex Basidiomycota, contain expanded novel kinase families termed FunK1 and SRPKL, which have been described only recently (Martinez, et al., 2012; Stajich, et al., 2010). The overview of the fungal region also helps to define the distribution of the TKL-group of kinases, which is present in earlier branching fungi and appears to have been lost on the branch giving rise to the Ascomycota.

The kinome of the Oomycete *Saprolegnia parasitica* is one of the largest observed. Many of these kinases are predicted to be membrane proteins in the TKL family, suggesting that they have roles in host interactions during the complex life cycle of this organism (Rays Jiang, personal communication). For several *Saprolegnia parasitica* kinases Kinannote reports that the best reference BLAST hits are to TK-group kinases, lending phylogenetic support to reports of TK-group kinases in *Phytophthora infestans* (Judelson, et al., 2010). The presence of these sequences in Oomycetes suggests an ancient origin for a group once thought to be solely metazoan.

# Supplemental Figure S1


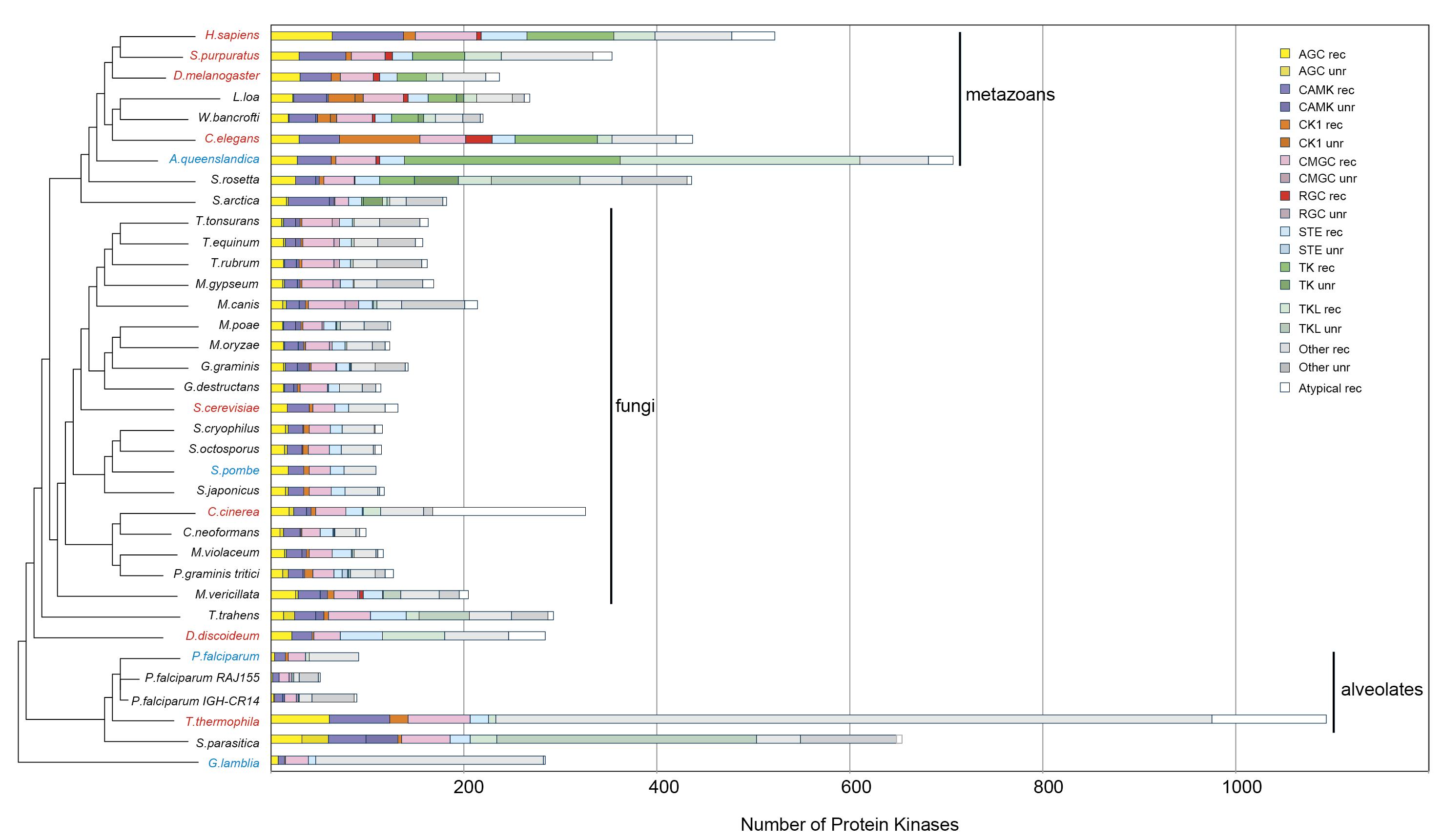


**Supplemental Figure S1**. Selected kinomes annotated by Kinannote, or a combination of Kinannote and manual curation in genomes released by the Broad Institute, shown in context with reference kinomes. The names of species with kinomes annotated by Kinannote are shown in black. The names of species with reference kinomes used for classification or ROC analysis are shown in red and blue, respectively. Protein kinases are classified into major groups (Hanks and Hunter, 1995), which are indicated by color (see key). Kinases that could not be fully classified are potentially novel, and are indicated using a darker shade of the group color.

#

# References

Goldberg, J.M.*, et al.* (2006) The dictyostelium kinome--analysis of the protein kinases from a simple model organism, *PLoS genetics*, **2**, e38.

Hanks, S.K. and Hunter, T. (1995) Protein kinases 6. The eukaryotic protein kinase superfamily: kinase (catalytic) domain structure and classification, *FASEB journal : official publication of the Federation of American Societies for Experimental Biology*, **9**, 576-596.

Judelson, H.S. and Ah-Fong, A.M. (2010) The kinome of Phytophthora infestans reveals oomycete-specific innovations and links to other taxonomic groups, *BMC genomics*, **11**, 700.

Manning, G., *et al*. (2011) The minimal kinome of Giardia lamblia illuminates early kinase evolution and unique parasite biology, *Genome biology*, **12**, R66.

Martinez, D.A., *et al*., (2012) Comparative Genome Analysis of Trichophyton rubrum and Related Dermatophytes Reveals Candidate Genes Involved in Infection, *mBio*, **3**.

Stajich, J.E. *et al*., (2012) Insights into evolution of multicellular fungi from the assembled chromosomes of the mushroom Coprinopsis cinerea (Coprinus cinereus), *Proceedings of the National Academy of Sciences of the United States of America*, **107**, 11889-11894.
