# Supplementary material for: Kinannote, a computer program to identify and classify members of the eukaryotic protein kinase superfamily
Source: Bioinformatics. 2013 Jul 31;29(19):2387–94. doi: 10.1093/bioinformatics/btt419 (PMC3777111; doi:10.1093/bioinformatics/btt419)
Supplement: Supplementary Data [file supp_btt419_suppl_data.zip › Supplemental_File_S1.tar!/Kinannote_1.0/doc/Kinannote_1.0_documentation.pdf]

Kinannote Documentation (5/17/2013)

1. Definition

Kinannote is a program that identifies and classifies protein kinases of the eukaryotic protein kinase superfamily, in a protein fasta file provided by the user, which may contain a whole proteome or a single sequence. It identifies kinases using a hidden Markov model, and classifies them using a BLAST search against of locally curated version of KinBase from www.kinase.com.

Authors: Jonathan Goldberg, Allison Griggs, and Janet Smith

2. Brief installation, usage and output statements

Download Kinannote from http://sourceforge.net/projects/kinannote/
Extract Kinannote (tar –xvf Kinannote_1.0.tar), cd Kinannote
Set up Kinannote: perl setup_kinannote.pl (follow prompts)
Usage: perl Kinannote_1.0.pl <options> <protein.fasta>

For metazoans and their close relatives:
perl Kinannote_1.0.pl **–m** –c <comment> <protein.fasta>

For non-metazoans:
perl Kinannote_1.0.pl –c <comment> <protein.fasta>

*The presence/absence of the '-m' flag is important for appropriate classification of kinases of the TK group, as described below*.

Output: Kinannote writes several files, including a file named <input_filename>.run_summary, where <input_filename> is the prefix of the input fasta file. This file contains names and brief descriptions of the other files written by Kinannote, and other information about the run.

3. Contents:


1. Definition
2. Brief usage and output statements
3. Contents
4. Dependencies
5. Installing and testing Kinannote
6. Running Kinannote
7. Troubleshooting
8. Kinannote's output files
9. Modules and reference data accompanying Kinannote
10. Interpreting results provided by Kinannote
11. Citing Kinannote


4. Dependencies:

Kinannote is a perl script tested with perl v5.12.3 and v5.10.1. Kinannote is launched by a unix shell command, and has the following dependencies:

1. python 2 (present on most unix systems, available from www.python.org). Tested on versions 2.4.3 and 2.7.1.
2. blastall from BLAST 2.2.24 release (available at http://mirrors.vbi.vt.edu/mirrors/ftp.ncbi.nih.gov/blast/executables/release/2.2.24)
3. hmmsearch from HMMER 2.3.2 release (available at http://hmmer.janelia.org/software/archive)

## 5. Installing and testing Kinannote

### Download and installation

1. Download Kinannote_1.0.tar from http://sourceforge.net/projects/kinannote/ and place it in a location of your choice on your system.
2. Enter the following commands in a unix shell:
   a. cd to the directory containing Kinannote_1.0.tar
   b. tar –xvf Kinannote_1.0.tar
   c. rm Kinannote_1.0.tar
   d. cd Kinannote_1.0
   e. perl  setup_kinannote.pl
      i. Follow prompts from the setup script
      ii. If appropriate versions of blastall and hmmsearch are not found, locate or install them, rerun setup_Kinannote.pl and provide paths to them at the appropriate prompts.
      iii. If the setup script reports that installation is successful, proceed to testing.

### Testing

1. From the Kinannote_1.0 directory
2. cd test
3. The test directory contains the file spombe.fasta, which contains the Schizosaccharomyces pombe proteome from http://www.broadinstitute.org/annotation/genome/schizosaccharomyces_group/MultiDownloads.html
4. Enter the command:

```
perl ../Kinannote_1.0.pl -c Spom spombe.fasta
```

Kinannote will run for about five minutes. Installation is confirmed by the following standard output:

```
Done!

The number of uniquely-identified sequences in spombe.fasta is 5142
The number of ePKs in spombe.fasta is 109: ePKs comprise 2.120 % of this proteome
The fraction of core ePK families found in this proteome is 0.938 (1 is best)
A list of hits with standardized names may be found in spombe.names
A summary of this kinannote run may be found in spombe.run_summary
```

## 6. Running Kinannote

General form of command, issued from a unix-like shell:

```
perl Kinannote_1.0.pl <options> <protein.fasta>
```

Protein fasta file:

The headers of protein fasta files analyzed by Kinannote file must contain unique sequence identifiers followed by spaces. If non-unique headers are provided, Kinannote will use the sequence associated with the last occurrence of the identifier.

Options:

Common options

1. **<-m> Treat the proteome as metazoan or a close relative thereof.**

   The presence of true TK-group kinases in non-metazoan kinomes has not been well established. Identification of a TK group kinase outside the metazoan would require user confirmation. At the Broad Institute, Kinannote is most often run on non-metazoans; its default behavior, therefore, does not allow classification to the TK group. If a non-metazoan sequence appears to be most similar to TK group kinases, the following comment is written to the <input_prefix>.kinannote_outfile and <input_prefix>.draft_kinome output files: 'best blast hit is TK'.

   *It is essential that the '-m' flag be included for appropriate classification of TK group kinases in metazoans and their close relatives.*

2. <-s> The input fasta is not a whole proteome. Skip core kinome analysis.
3. <-c comment> Populate columns in the <input_prefix>.kinannote_outfile and <input_prefix>.class_depth output files with a comment from the user. This comment is optional but is useful the need arises to concatenate Kinannote output files.

Options for parameter testing

1. <-a tag> Add a tag to the name of output files, which will appear after the input_file prefix. This tag allows distinction between runs on the same fasta file with different parameter settings.
2. <-p filename> This option allows Kinannote to read a parameters file. Defined parameters in this file overwrite built-in parameters; built in parameters not defined in this file are retained. Each Kinannote run produces a parameters file, <input_prefix_tag>.parameters_out. This file may be edited and used as an input parameter file with the –p option.

What to expect

Kinannote usually takes about five minutes to run, and produces standard output like:

Done!

The number of uniquely-identified sequences in spombe.fasta is 5142
The number of ePKs in spombe.fasta is 109: ePKs comprise 2.120 % of this proteome
The fraction of core ePK families found in this proteome is 0.938 (1 is best)
A list of hits with standardized names may be found in spombe.names
A summary of this kinannote run may be found in spombe.run_summary

If this does not occur, please see Troubleshooting

7. Troubleshooting

If Kinannote runs in seconds, not minutes, or the if numbers in the screen summary don't make sense (lots of 'zeros'), then something went wrong. If this occurs:

1. Check the command used to launch Kinannote and the input fasta file.
2. If you were using the –p option, try the run again using built-in parameters.
3. Rerun on spombe.fasta from the test directory provided with Kinannote.
4. Rerun setup_Kinannoate.pl, or retrieve a fresh copy from http://sourceforge.net/projects/kinannote/.
5. If troubleshooting fails please contact me at jmgold@broadinstitute.org.
6. If Kinannote inappropriately classifies TK group kinases into the TKL group, rerun Kinannote with using the '-m' flag.

8. Files generated by Kinannote:

Files generated by Kinannote

| File name, preceded by the prefix of the input fasta file | Order Written | Contents |
|---|---|---|
| .run_summary | 10 | Summary of the new draft kinome and outfiles |
| .draft_kinome | 7 | Accession numbers, classifications, scores, classification levels and classifications of kinases, starting point for in-depth kinome curation |
| .names | 6 | Accession numbers and classification-based gene product names for new draft kinome |
| .merged_classifications | 8 | Phylogenetic profile of the new draft kinome in the context of the Dd, Sc, Ce, Dm and Hs kinomes[1], reports potential missing core kinases for hole-filling and proteome health |
| .parameters_out | 9 | Parameters used by Kinannote, this table may be used as input for subsequent runs and edited |
| .hmmsearch_outfile | 1 | Results of an hmmersearch run of the input proteome against the protein kinase HMM |
| _candidates.fa | 2 | Fasta file of residues from candidate sequences that matched the search HMM |
| _candidates_BLAST.outfile | 3 | Output of a BLAST run of candidates.fa against the curated kinase database |
| .kinannote_outfile | 4 | Accession numbers, scores, and classifications of the new draft kinome, input for modules |
| .kinase_summary | 5 | Number of kinases per classification category in the new draft kinome, input for the phylogenetic profile in .merged_classifications |

1. Reference kinomes for phylogenetic analysis: Dd, *Dictyostelium discoideum*; Sc, *Saccharomyces cerevisiae*; Ce, *Caenorhabditis elegans*; Dm, *Drosophila melanogaster*; Hs, *Homo sapiens*

9. Modules and reference data accompanying Kinannote

1. Kinannote/modules/KinannoteModules12.pm: modules that manage kinome summary, comparative kinomics and gene product naming.
2. Kinannote/lib/discovery.hmm: eukaryotic protein kinase hidden Markov model built in HMMER 2, based on an alignment of the Dictyostelium kinome (Goldberg, J. M., G. Manning, et al. (2006). "The dictyostelium kinome--analysis of the protein kinases from a simple model organism." PLoS Genet **2**(3): e38.)
3. Kinannote/lib/Pkinase_scoring_matrix.discTrans: protein kinase conserved motif matrix
4. Kinannote/lib/blast/KDB_20111223b: BLAST database of locally updated curated kinases from kinase.com
5. Kinannote/lib/( dicty.txt, fly.txt, human.txt, worm.txt, yeast.txt): kinome summaries from species with curated kinomes for placing the current automated kinome into phylogenetic context and identifying potentially missed core kinases.

10. Interpreting results provided by Kinannote

1. *Fully and partially classified kinases*. The names and classifications of fully and partially classified kinases from the names and draft_kinome files produced by Kinannote may be used as terms for searches of the biomedical literature, and for searches of kinase-specific resources such as KinBase (www.kinase.com), ProKinO (http://vulcan.cs.uga.edu/prokino), the Protein Kinase Resource (http://pkr.genomics.purdue.edu/), KinG (http://king.mbu.iisc.ernet.in) and PhospoSite (http://www.phosphosite.org). The results of such searches are often broader than searches based on specific kinase names. The classifications provided by Kinannote also facilitate comparing kinomes of different species.
2. *Members of potentially novel kinase families*. Novel and species-specific kinases, and kinases whose gene models may have problems, do not cluster well with kinases from known families. Such sequences are identified in files produced by Kinannote by the name 'serine/threonine protein kinase' or by absence of classification beyond the group level. The draft_kinome table contains a column with information about the depth to which sequences could be classified: classification depths of '0' and '1' indicate that the kinases are unclassified, or classified only to the group level, respectively, and may be members of novel families. Further curation is required to confirm the novelty of sequences, to determine relationships between them, and to identify relatives in non-reference species.
3. *Potentially novel protein kinase-like (PKL) sequences*. PKLs differ from typical protein kinases, but share structural and sequence motifs involved in catalysis and binding. Candidate PKLs are identified in the draft_kinome table by the tags 'protein kinase subdomain-containing protein' and 'twilight' in the name and classification_note columns, respectively. Twilight hits should not be interpreted as likely PKLs; rather, they are provided to identify seed sequences for investigation of new families that may occur in some species.

11. Citing Kinannote

http://sourceforge.net/projects/kinannote
